# Vaccination status, favipiravir, and micronutrient supplementation roles in post-COVID symptoms: A longitudinal study

**DOI:** 10.1371/journal.pone.0271385

**Published:** 2022-07-21

**Authors:** Bumi Herman, Martin Chi-sang Wong, Pramon Viwattanakulvanid

**Affiliations:** 1 College of Public Health Sciences, Chulalongkorn University, Bangkok, Thailand; 2 Clinical Epidemiology, Research Development & Publication, Faculty of Medicine, Hasanuddin University, Makassar, Indonesia; 3 School of Public Health and Primary Care (JCSPHPC) at the Chinese University of Hong Kong, Shatin, Hong Kong; 4 The Chinese Academy of Medical Sciences and the Peking Union Medical College, Beijing, China; 5 The School of Public Health, The Peking University, Beijing, China; The 8th Medical Center of PLA General Hospital, CHINA

## Abstract

**Introduction:**

Post-COVID symptoms are the new concern in the COVID-19 pandemic, where recovered patients experience residual symptoms affecting their quality of life. Therefore, it is imperative to evaluate the role of complete vaccination, prescribed medication, and micronutrients during COVID episodes in the occurrence of post-COVID symptoms.

**Method:**

A longitudinal evaluation of Indonesia’s recovered COVID-19 patients was performed using the data collected from July 2021 and extracted in mid-February 2022. All participants were confirmed with a Real-Time Polymerase Chain Reaction test (PCR) and/or antigen test. This study collected demography and comorbidities information, symptoms and treatment of COVID-19, and collection of self-reported post-COVID symptoms every 30 days within 90 days after diagnosis/onset. Exposures of interest include vaccination status, Favipiravir administration, Vitamin C, Vitamin D, and Zinc. A Generalized Estimating Equation (GEE) was used to evaluate the longitudinal effect of exposures, presented with adjusted odds ratios and its 95% confidence interval.

**Results:**

A total of 923 participants (18.2% fully-vaccinated) were involved in the study, with 79.7% being non-hospitalized. Only 25.7% did not develop any residual symptoms within 90 days. Fatigue was the most reported post-COVID symptom in each measurement time (39.5%, 16.3%, and 7.3%). Full-vaccination was effective against chronic cough (aOR 0.527, 95% CI 0.286–0.971), chronic headache (aOR 0.317, 95% CI 0.163–0.616), and chronic arthritis (aOR 0.285, 95% CI 0.116–0.703). The combination of micronutrient supplementations and Favipiravir gave no significant effect on all post-COVID symptoms. However, early initiation of Favipiravir and delaying vitamin D administration were associated with arthritis.

**Conclusion:**

Full vaccination of COVID-19 prevents the disease and the development of residual symptoms when infected with SARS-COV-2. Hence, it is crucial to reconsider the prescription of micronutrient supplementation or adjust the dose of Favipiravir in the current guideline.

## Introduction

The global pandemic of Coronavirus Disease 2019 (COVID-19) records more than 440 million infections in March 2022, with 5.9 million deaths. In Southeast Asia, 56 million were contracted by SARS-COV-2, with 764.410 fatalities. Indonesia is heading the region with 5.7 million cases and 149,596 mortalities [1]. COVID-19, particularly with its variant of concern, introduces a burden to the health care system due to its rapid transmission, systemic clinical manifestations, and possible long-term effect, as 30–50% of people may experience residual symptoms within one month [2, 3]. It is crucial to address the later issues as many survivors have the quality of life deterioration. The term post-COVID appears as "Signs and symptoms developed during or following a disease consistent with COVID-19 and which continue for more than four weeks but are not explained by alternative diagnosis" [4]. Many classifications have been introduced based on the duration, including acute, long, and persistent post-COVID symptoms [5]. A 7-month longitudinal study combining data from different countries shows systemic symptoms, including Fatigue and organ-specific symptoms, are found in COVID survivors [6]. Indonesia was included in this study, although a national study is needed to describe a more comprehensive situation.

Post-COVID symptoms are assumed to result from the Central nervous system (CNS) perturbation and systemic inflammation. The "ascending dissemination of virus" theory may explain why some survivors develop CNS disturbance, although the possibility of hematogenous dissemination could not be eliminated [7]. Furthermore, fragments of SARS-COV-2 can be found in many parts of the body later after the infection resolve, which seems unclear whether it is linked to post-COVID [8]. Therefore, it is imperative to prevent or reduce post-COVID symptoms by applying appropriate medication that interferes with these mechanisms.

### Vaccination as first-line management

Vaccination against COVID-19 is vital to boost immunity against SARS-COV-2. The stimulated T-lymphocytes and B-lymphocytes cells reduce the probability of virus replication by inhibiting the virus to the host’s receptor or initiating the cytotoxic mechanism of infected cells [9]. Therefore, the inhibition of virus replication by the immunity in vaccinated individuals possibly reduces the subsequent impact of infection and the long-term effects of COVID-19.

Indonesia selected an inactivated vaccine, administered 28 days, or a viral vector vaccine 8–12 weeks apart as the prime vaccine. The procurement of mRNA (given in 21 days) vaccine in September 2021 strengthens the vaccination program, particularly for those who cannot receive the two vaccines. In January 2022, the government initiated the booster doses in response to several studies indicating vaccine efficacy waning. As of February 2022, The rate of fully-vaccinated people is less than 54%, and only 5% population received booster doses [10]. Therefore, it is essential to prove the possible benefit of vaccination in reducing disease severity and its long-term effect on increasing vaccination coverage, particularly for those questioning its benefit.

### Which medication is essential?

As a virus causes COVID-19, management of the disease solely depends on the symptoms and severity. Two critical things in COVID-19 management are preventing direct viral invasion and pro-inflammatory conditions. The current guidelines emphasize the prescription of antivirus and micronutrients to prevent further inflammation. Unfortunately, some treatments are no longer effective, including plasma convalescent [11]. The role of micronutrient supplementation remains questionable. Despite the conflicting results and lack of evidence, these micronutrients exist in current guidelines in Indonesia and other countries, and there is a need to justify the prescription of these supplements.

Antivirus for COVID-19 inhibits the replication of the virus on the cell hosts. Favipiravir is one of the antiviral drugs prescribed for COVID-19. This drug acts as an analog of purine in nucleic acid, where it is converted intracellularly into an active form that disrupts the RNA polymerase/RdRp [12]. Favipiravir reduces the viral shedding duration of SARS-COV-2 and the level of pro-inflammatory markers [13]. By preventing the replication of the virus, and direct injury of tissue, particularly in the CNS and other system organ, Favipiravir is hypothetically beneficial in preventing the long-term effect of COVID-19.

Micronutrient supplementation enlisted in the national guideline is vitamin C, Vitamin D, and Zinc. However, there is no specific dose associated with a better prognosis. A meta-analysis revealed heterogeneity in doses and administration of these micronutrients and no significant effect on mortality but the various effect on the need for intubation and length of stay [14]. Moreover, micronutrient supplementation is not free from side effects. For example, gastrointestinal disturbance and Hypercalcemia in Vitamin D toxicity were reported, although the incidence might be lower in a low dose of oral supplementation.

The current guideline recommends that these micronutrients be taken orally. However, oral supplementation depends on individual tolerability and may not provide maximum effect compared to intravenous administration. Therefore, it is fundamental to simplify the current guideline if these micronutrients have no significant protective effect on COVID severity and long-term effect.

### Objectives

This study aims to evaluate the effect of vaccination, Favipiravir, and micronutrient supplementation on post-COVID symptoms. It is assumed that vaccination and antiviral drug are beneficial for preventing and or reducing post-COVID symptoms, and micronutrient supplementation may have a very minimal role in post-COVID symptoms.

## Methodology

### Setting and study design

This study is a longitudinal survey of the Indonesian POST-COVID study initiated in July 2021. An online questionnaire gathered the demographic and clinical data of recovered COVID-19 patients from the whole of Indonesia. The recruitment of participants relied on the referral technique where the patients invited other family members or colleagues who recovered from COVID-19. Participants consented to submit supporting information, including a hospital résumé (if hospitalized) and an independent lab examination sought as part of their COVID-19 standard treatment. The participants knew that the researchers would contact them for consecutive follow-ups for various post-COVID symptoms. This study involved all participants who completed the longitudinal follow-up until mid-February 2022.

### Participants eligibility

All participants should be diagnosed with COVID-19 with quantitative Real-Time Polymerase Chain Reaction/qRT-PCR or other Nucleic Acid Amplification Test/NAAT [15] of the nasopharyngeal sample. In addition, participants screened with antigen tests approved by Indonesia’s Ministry of Health were eligible. Those people screened with antibody test should be confirmed with qRT-PCR or antigen test, particularly those who demonstrated positive results of IgM antibody. These patients should recover less than 30 days after the onset (or after diagnosis day for those without symptoms). Definition of recovery depends on the severity of the case where those people with home isolation could end their isolation by undergoing two repeated qRT-PCR or completing the recommended duration (ten days for asymptomatic cases, or ten days after the onset plus three days without fever and respiratory symptoms) following the World Health Organization guideline [16]. In moderate and severe cases, physician discretion based on the resolution of symptoms and negative qRT-PCR results is essential to determine the recovery status. It is necessary to exclude those who recovered more than 30 days, as this study focuses on the post-covid symptoms that start as early as 30 days after the onset/Diagnosis. The exclusion was made to those unable to do the follow-up, those with suggestive reinfection, and those who also received other antivirus medication (Oseltamivir, Molnupiravir, and Remdesivir). Those participants who received the other antivirus were severe cases that required hospitalization for more than 30 days. Although Oseltamivir is given to those with mild symptoms, it is no longer listed as first-line therapy for COVID-19 in Indonesia. As for Molnupiravir, none of the participants in the cohort received this new medication. No exclusion of participants was made based on the presence of comorbidities or chronic disease or the age of participants.

### Variables

This study gathered data on three distinct sorts of data. First, demographic variables consist of the age at diagnosis or onset, sex, occupation (medical or non-medical), level of education, island, type of living region (from rural to urban), daily activity (working outside or staying at home), with whom they live during COVID-19, and health insurance coverage.

Additionally, data on current health status and comorbidities, such as body mass index during COVID-19, smoking and alcohol consumption within three months prior to diagnosis, moderate physical activity, and the presence of other diseases (controlled or uncontrolled) were also collected.

Collection of COVID-19 symptoms and treatment data was conducted, including the details of diagnosis method, duration of symptoms, prescribed medication including advance therapy, and the status of treatment (home isolation, hospitalization, or referred cases).

### Tools

The questionnaire was developed in response to post-COVID-19 symptoms and potential confounding variables. ORF1-ab, Nucleocapsid, RdRP (RNA-dependent RNA Polymerase), and Spike genes linked with SARS-COV-2 infectivity are used for SARS-COV-2 detection through qRT-PCR [17]. The lateral flow/antigen test is acceptable for diagnosis. However, it should be done in an accredited laboratory using at least 80% sensitivity and 97% specificity kit [18].

### Exposures

In this cohort, the researchers would like to explore the effect of Favipiravir, micronutrient supplementation of Zinc, Vitamin C, and Vitamin D. Additional interest includes the effect of complete vaccination on the prevention of selected post-COVID-19 symptoms.

The national guideline recommends oral Favipiravir administration of 1600 mg /12 hours on the first day, followed by 600 mg every 12 hours on days 2–5 [19]. This regimen is given to those who can take it orally, regardless of disease severity. However, the researchers noticed that this recommended dose is lower than others [20]. Furthermore, physician consideration plays a critical role in Favipiravir prescription. The presence of other diseases, including renal failure [21], pregnancy [22], hypersensitivity, and the duration of onset or whether the symptoms have resolved entirely without any specific interventions, are some of the reasons why the Favipiravir prescription may vary between individuals.

Oral vitamin C and Vitamin D are listed in the National Guideline. However, the doses differ from the previous guideline. The latest guidelines recommend 500 mg of vitamin C per 12 hours followed by Vitamin D 1000 IU daily for 14 days. Zinc is also given as low as 20 mg/daily. These micronutrient preparations are prescribed with antivirus or purchased over the counter as a single multivitamin tablet. The researchers were aware of the heterogeneity of micronutrient supplementation. Therefore, this study set a limit of vitamin C as 500 mg twice daily or 1000 mg daily and Vitamin D at least 800 IU daily in the questionnaire following the previous edition of the guideline (before January 2022).

This study also compares the different vaccination statuses. The exposure group was fully-vaccinated and contracted SARS-COV-2 at least 14 days after receiving the second dose. The comparison group was those who only received one dose or unvaccinated individuals. Finally, the difference in post-COVID proportion between groups would be observed.

### Outcomes

The self-reported symptoms are the outcomes of this study. These include fatigue, chronic headache, brain fog, alteration in concentration, chronic cough, joint pain (arthritis), diarrhea, and hair loss which represent the systemic inflammation effect of COVID-19. Unfortunately, unlike depression or anxiety that a specific health questionnaire can measure, there is no appropriate questionnaire that reliably screens and diagnoses the symptoms of post-COVID-19. For example, the Short Fatigue Questionnaire is reliable for severe fatigue [23]; however, there is limited information on the degree of post-COVID-19 fatigue severity, and if it is mild, some questionnaires might be unable to screen the mild Fatigue after COVID-19. In addition, some clinical questionnaires also have overlapping questions with other symptoms. Therefore, conducting specific clinical measurements through the questionnaire will create a "survey fatigue," which will lead to a lower response rate. Furthermore, this study excluded outcomes that should be confirmed with clinical examination, such as chest pain and palpitations, as it needs electrocardiogram confirmation.

On the follow-up questionnaire, the participants were asked several questions preceded by this sentence, "Compared to the time before you have COVID-19, At this time, do you experience any of these symptoms that occur only after recovering from COVID?" and ended by this statement, "Did you consult with the physician and confirmed that there are no other underlying reasons of why these symptoms still occur?" This study asked the participants every 30 days for a total of 90 days after their first day of onset or diagnosis. Hence, each participant would have three repeated measurements of seven outcomes. The outcomes were classified into binary responses (yes or no).

### Study size and possible bias

The sample size was estimated using the difference in participants’ proportion who developed post-COVID-19 symptoms. Several assumptions were applied, including the superiority effect of receiving all regiments and fully vaccinated (one tail hypothesis), type I error of 5%, and power of study of 80%. Among seven outcomes, fatigue was the most reported symptom, followed by chronic dry cough and diarrhea, according to one study of Long-COVID [6]. This study selected chronic dry cough as the outcome for sample size estimation with a prevalence of 66%, considering that physiological alteration initially occurs in the respiratory tract. Assume that people who received all interventions (Favipiravir, Zinc, Vitamin C, and Vitamin D) were around 25% of the total cohort, and interventions would have an absolute reduction of 10%; the estimated total sample was 922 respondents.

The ideal design to evaluate the effectiveness of treatment is using a randomized control trial (RCT) or making a quasi-design subset from the cohort. However, this study has multiple exposures, and it was not feasible to assign the participants from the cohort using the matching method (propensity matching, case-control matching, or weight matching) into equal proportion, mimicking the clinical trial. Furthermore, this study wanted to address the combined effect of therapy on selected post-COVID-19 outcomes. Therefore, the researchers decided to analyze the data using the longitudinal cohort approach, which has a lower evidence level than quasi-design or RCT.

The researchers also identified other potential biases, such as the recall bias of information and procedural bias (including the heterogeneous doses of medication and adherence to medication). Recall bias, notably in the data on symptoms, was unavoidable, especially for people who underwent home isolation. On the other hand, telemedicine could verify the accuracy of the symptoms and the medication adherence, as home isolation requires patients to observe and monitor their symptoms. The researchers underlined this point in the consent form by instructing respondents to complete the form using their telemedicine observation chart. Aside from that, variation in the length of the Favipiravir and other medications prescription also occurred. Some people may not receive the medications on the day of diagnosis, and ignoring this will introduce statistical bias as these variables are time-varying covariates. An adjustment was made accordingly in the statistical analysis.

### Quantitative variables

Age, body mass index, and duration of COVID-19 symptoms were not discretized. As the time of receiving medication differed, this study classified the time into <24 hours, 24–72 hours, >72 hours, and not receiving any medication to accommodate the time-varying exposure.

### Statistical analysis

This study did not impute the missing data and did the listwise deletion method to incomplete responses. Descriptive statistics and normality tests were executed to assess and determine the distribution of continuous data. The baseline information was presented according to the occurrence of any post-COVID symptoms within 90 days after onset/diagnosis (overall event).

Since many variables were collected, there would be a possibility that some variables might violate the collinearity assumption. Therefore, the researchers assumed that one symptom may be linked to other symptoms and could represent the severity of the disease and treatment. Bivariate tests were conducted to select the parameter representing other variables and simplify the final analysis.

The first evaluation of exposure variables was conducted using binary logistic regression. There were two groups of participants according to post-COVID symptoms within 90 days after onset/diagnosis. Each intervention was analyzed and adjusted by several factors associated with the overall event of post-COVID in a hierarchical sequence. The final model combined four treatments and vaccination status with adjustment of confounders. The measurement of association was presented as an adjusted odds ratio (aOR) with a 95% Confidence Interval.

This study also accounted for the time-varying outcomes. Some people may experience the symptoms, and they diminish over time. Therefore, it is crucial to address temporality by conducting a time-to-event analysis. Several studies of Long COVID asked the participants to recall the exact time of post-COVID symptoms to fit the survival analysis. The questionnaire assessed the outcome at a specific time (30, 60, and 90 days). This study introduced two different methods, treating the outcome at the time of measurement (considering that the symptoms may not continuously appear at every measurement time) and using an interval-censored response, as the specific time when the symptoms appear was unknown. Hence, the repeated measure analysis using Generalized Estimating Equation (GEE) was more appropriate to analyze the effect of these exposures, particularly in an unbalanced proportion of the cohort.

The GEE assessed the effect of each medication and intervention and the combined medication. Several assumptions of GEE were also assessed. The robust estimator calculated the covariance matrix of the model. The response was treated as binary with a logit link function (outcome at time-point) and a complementary log-log link function (interval-censored response). This study estimated the Quasi Likelihood Under Independence Model Criterion (QIC) value to select the appropriate correlation matrix structure. Type III test of effect was conducted based on Wald Chi-Square. The adjusted odds ratio (aOR) confidence interval was 95%. Adjusted variables were added in hierarchical order in the final model.

### Ethical approval

The Research Ethics Review Committee for Research Involving Human Research Participants at Hasanuddin University approved this study (full-board review number 758/UN4.6.4.5.31/PP36/2021). Consent was gained from participants when they submitted data to the cohort. To protect the confidentiality, we de-identified, retained, and used the data appropriately. Any case requiring immediate care was referred to a specialist. This study is part of a clinical trial registered at clinicaltrials.gov NCT05060562.

## Results

### Baseline information

**Fig 1:**
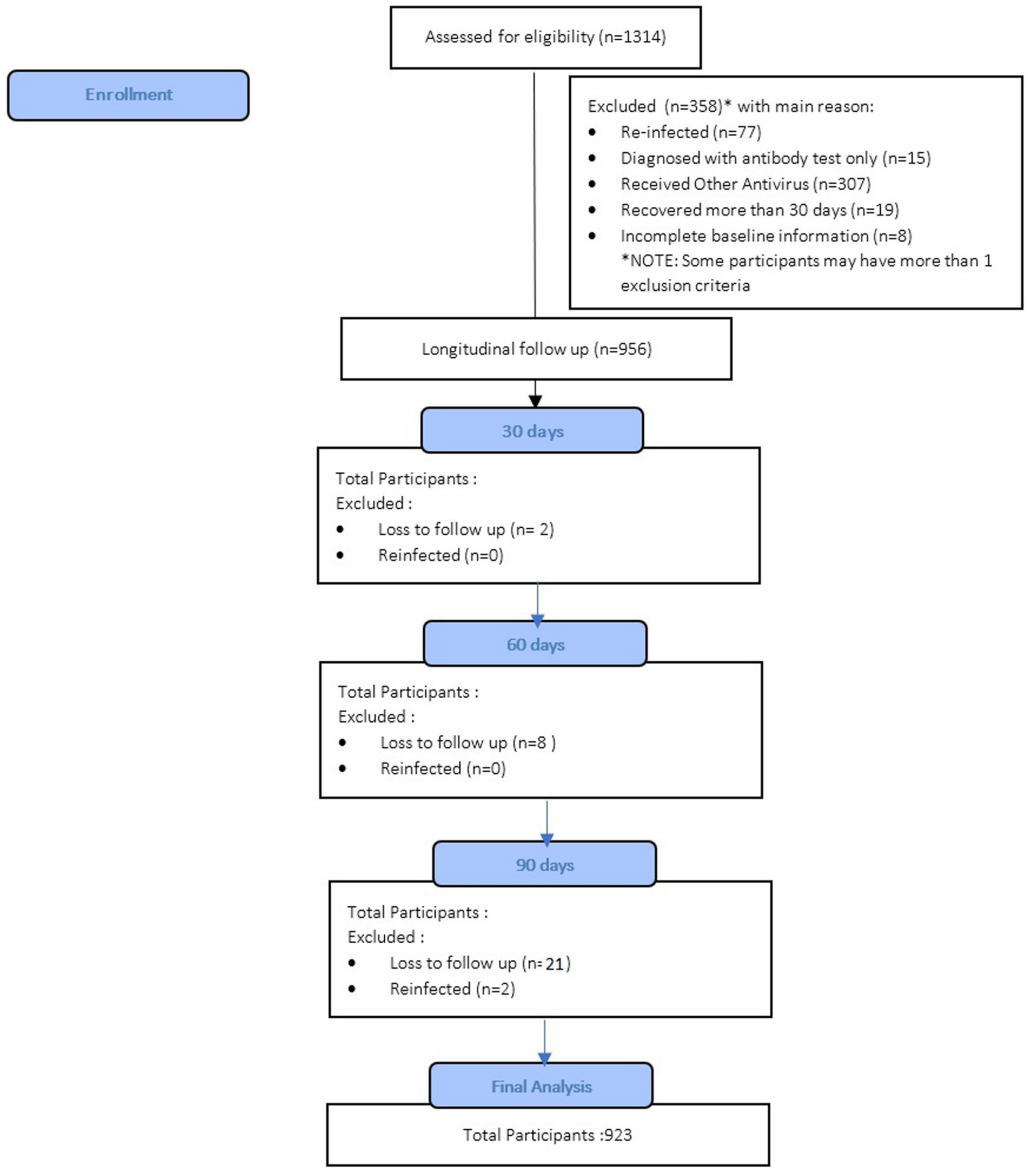
Flow diagram of the participant selection process (n = 923).

A total of 923 participants’ data was extracted from the primary cohort. The selection of participants is depicted in **(Fig 1)**. The average age was 32.77 (SD ± 10.04) years old and dominated by the female (59%), with the vast majority of cases detected through QRT-PCR only (45.5%). The QRT-PCR test also confirmed some cases that underwent an antigen test (22.4%), antibody test (0.3%), and both antibody and antigen test (1.4%). As 265 participants (28.7%) were screened with an antigen test, 1.6% of tested positive with an antibody test were confirmed by an antigen test. Medical personnel accounted for 26.5%, and only 40% of respondents live alone. The distribution of cases was found in Jawa (31.2%), followed by Kalimantan (24.2%), Sumatera (18.8%), Sulawesi (14.2%), Bali, and Nusa Tenggara (8.7%), whereas the least number of cases found in the eastern part of Indonesia (2.9%). Most participants came from the capital area (45.7%), lived with other people at their residence (60.0%), and still working or learning outside their residence (53.3%). Six hundred eighty-six participants (74.3%) reported at least one of the selected residual symptoms within 90 days after onset/diagnosis.

**Table 1** explains the baseline information according to post-COVID symptoms within 90 days after onset or diagnosis. In terms of demographic factors, there was a significant difference in the age where older age was associated with the post-COVID symptoms (p<0.001). Moreover, those who stayed alone were more likely to develop symptoms (crude Odds Ratio 1.62, 95% CI 1.19–2.22). However, there was no significant difference between the presence of diseases and the occurrence of any post-COVID symptoms. Regarding health behavior, smoking, physical exercise, and alcohol drinking were not associated with post-COVID symptoms.

**Table 1:** Characteristics of participants (n = 923).

Variables	Subset	Any residual symptoms within 90 days?		*P-value*
		Yes(%)	No(%)	
Sex	Female	403(73.9)	142(26.1)	0.752
	Male	283(74.9)	95(25.1)	
Occupation	Non Medical Personel	514(75.8)	164(24.2)	0.085
	Medical Personel	172(70.2)	73(29.8)	
Education Level	up to Diploma	371(75.4)	121(24.6)	
	Bachelor’s Degree and Graduate Level	315(73.1)	116(26.9)	
Island	Sumatera	141(81.0)	33(19.0)	<0.001*
	Jawa	213(74.0)	75(26.0)	
	Bali and Nusa Tenggara	60(75.0)	20(25.0)	
	Kalimantan	174(78.0)	49(22.0)	
	Sulawesi	78(59.5)	53(40.5)	
	Maluku and Papua	20(74.1)	7(25.9)	
Type of living area	Rural Area	185(77.4)	54(22.6)	0.341
	Urban not capital	196(74.8)	66(25.2)	
	Capital area	305(72.3)	117(27.7)	
Live alone or with someone else?	With family, Colleagues, or relatives	392(70.8)	162(29.2)	0.002*
	Alone	294(79.7)	75(20.3)	
Health Insurance	National Health Insurance	336(72.7)	126(27.3)	0.366
	Private Insurance	178(77.7)	51(22.3)	
	No / Currently Inactive	172(74.1)	60(25.9)	
Activity	Diverted to residence	309(71.7)	122(28.3)	0.087
	Mostly outside residence	377(76.6)	115(23.4)	
Hypertension	No/Unknown	635(74.0)	223(26.0)	0.469
	Yes, controlled	33(82.5)	7(17.5)	
	Yes, uncontrolled	18(72.0)	7(28.0)	
Diabetes Mellitus	No/Unknown	659(74.1)	230(25.9)	0.729
	Yes, controlled	13(76.5)	4(23.5)	
	Yes, uncontrolled	14(82.4)	3(17.6)	
Asthma	No/Unknown	669(74.9)	224(25.1)	0.069^
	Yes, controlled	14(56.0)	11(44.0)	
	Yes, uncontrolled	3(60.0)	2(40.0)	
HIV	No/Unknown	684(74.3)	237(525.7)	1.000^
	Yes, controlled (undetectable)	2(100)	0 (0)	
COPD	No/Unknown	679(74.2)	236(25.8)	0.688^
	Yes, controlled	7(87.5)	1(12.5)	
Cancer/Malignancy	No / Unknown	682(74.2)	237(25.8)	0.577^
	Yes	4(100)	0 (0)	
Dyspepsia Syndrome	No / Unknown	404(75.4)	132(24.6)	0.390
	Yes	282(72.9)	105(27.1)	
Heart Disease	No / Unknown	676(74.1)	236(25.9)	0.306^
	Yes	10(90.9	1(9.1)	
Stroke	No / Unknown	685(74.4)	236(25.6)	0.488^
	Yes	1(50)	1(50)	
Mental Health Disorder	No / Unknown	669(74.4)	230(25.6)	0.692
	Yes	17(70.8)	7(29.2)	
Autoimmune Disease	No / Unknown	683(74.5)	234(25.5)	0.180^
	Yes	3(50)	3(50)	
Kidney Disease	No / Unknown	684(74.4)	235(25.6)	0.273^
	Yes	2(50)	2(50)	
Liver Disease	No / Unknown	683(74.3)	236(25.7)	1.000^
	Yes	3(75.0)	1(25.0)	
Smoking Status	Never	634(74.7)	215(25.3)	0.405
	Ever/Currently Smoking	52(70.3)	22(29.7)	
drink alcohol at least three months prior to diagnosis	No	657(74.7)	223(25.3)	0.290
	Yes	29(67.4)	14(32.6)	
Frequency of doing moderate exercise in a week prior to diagnosis	>3 times per week	57(67.1)	28(32.9)	0.254
	2–3 times per week	104(73.8)	37(26.2)	
	1 or less per week	525(75.3)	172(24.7)	
Vaccination status before infected	Received one dose/unvaccinated	579(76.7)	176(23.3)	<0.001
	Fully vaccinated >14 days	107(63.7)	61(36.3)	
Age	Mean ± Std Deviation	33.98±10.18	29.28±8.75	<0.001
BMI	Mean ± Std Deviation	23.19±4.60	23.77±4.78	0.169

^Fischer Exact. Other categorical variables tested with Chi-Square

All continuous data were tested with Mann Whitney

* Significant at *P-value* < 0.05

As 168 (18.2%) people who were fully vaccinated with two doses of inactivated viral vaccine were infected more than 14 days after receiving their second dose with an average of 85.9 days. Three people were infected less than 14 days after the second dose of inactivated viral vaccine, and one person was infected less than 14 days after receiving the first dose of the inactivated vaccine. Forty-one people were inoculated with one dose of vaccine (17 with viral vector and 24 with inactivated viral vaccine). Furthermore, 713 (77.2%) were unvaccinated at diagnosis. Those infected more than 14 days after receiving the second dose were protected from post-COVID symptoms (crude Odds Ratio 0.53 95% CI 0.37–0.76). Fatigue was the most reported post-COVID symptom in each measurement time (39.5%, 16.3%, and 7.3%), followed by chronic headache and diarrhea (Table 1B in **S1 Table**).

A total of 736 participants were treated at home (79.7%). Longer duration of symptoms and longer days of treatment were observed in those people who developed any post-COVID symptoms (p<0.001), except diarrhea (p = 0.113). In addition, antipyretic, Azithromycin, cough medication (all p<0.001), and anticoagulant were connected to post-COVID symptoms. However, Zinc, Vitamin D, Vitamin C, and Favipiravir had an insignificant effect on post-COVID symptoms (p>0.05). **Table 2** elucidates the symptoms and treatment received during the COVID episode.

**Table 2:** Association between residual symptoms and received Covid-19 treatment within 90 days.

Variables	Subset	Any residual symptoms within 90 days?		*P-value*
		Yes(%)	No(%)	
Antipyretic	No Fever	10(24.4)	31(75.6)	<0.001*
	<24 hours after the fever appear	387(77.4)	113(22.6)	
	24–72 hours after the fever appear	127(79.4)	33(20.6)	
	>72 hours after the fever appear	12 (50.0)	12 (50.0)	
	Not receiving any treatment	34 (47.2)	38 (52.8)	
Anti-inflammation	<24 hours after onset	148(71.5)	59(28.5)	0.357
	24–72 hours after onset	83(72.8)	31(27.2)	
	>72 hours after onset	48(82.8)	10(17.2)	
	Not receiving any treatment	407(74.8)	137(25.2)	
Azithromyicin	<24 hours after diagnosis	271(80.2)	67(19.8)	<0.001*
	24–72 hours after Diagnosis	215(77.3)	63(22.7)	
	>72 hours after Diagnosis	103(83.1)	21(16.9)	
	Not receiving any treatment	97(53.0)	86(47.0)	
Cough medication	<24 hours after onset	216(76.1)	68(23.9)	<0.001*
	24–72 hours after onset	215(79.9)	54(20.1)	
	>72 hours after onset	95(78.5)	26(21.5)	
	Not receiving any treatment	160(64.3)	89(35.7)	
Vitamin C 500 mg daily	<24 hours after onset / diagnosis	322(74.4)	111(25.6)	0.373
	24–72 hours after onset / diagnosis	172(77.1)	51(22.9)	
	>72 hours after onset / diagnosis	90(68.7)	41(31.3)	
	Not receiving any treatment	102(75.0)	34(25.0)	
Vitamin D at least 800 IU daily	<24 hours after onset / diagnosis	259(73.2)	95(26.8)	0.727
	24–72 hours after onset / diagnosis	151(75.5)	49(24.5)	
	>72 hours after onset / diagnosis	76(78.4)	21(21.6)	
	Not receiving any treatment	200(73.5)	72(26.5)	
Zinc	<24 hours after onset / diagnosis	242(76.1)	76(23.9)	0.082
	24–72 hours after onset / diagnosis	141(74.6)	48(25.4)	
	>72 hours after onset / diagnosis	72(82.8)	15(17.2)	
	Not receiving any treatment	231(70.2)	98(29.8)	
Favipiravir	<24 hours after diagnosis	111(76.6)	34(23.4)	0.653
	24–72 hours after Diagnosis	89(74.8)	30(25.2)	
	>72 hours after Diagnosis	53(79.1)	14(20.9)	
	Not receiving any treatment	433(73.1)	159(26.9)	
Anticoagulant	<24 hours after diagnosis	12(85.7)	2(14.3)	0.032*
	24–72 hours after diagnosis	7(77.8)	2(22.2)	
	>72 hours after Diagnosis	16(76.2)	5(23.8)	
	Not receiving any treatment	651(74.1)	228(25.9)	
Received oxygen supplementation	No	619(73.9)	219(26.1)	0.531
	Less than 3 days	36(76.6)	11(23.4)	
	More than 3 days	31(81.6)	7(18.4)	
received plasma convalescent	No	678(74.3)	235(25.7)	1.000^
	Yes	8(80)	2(20)	
Unit of care	Home Isolation	535(72.7)	201(27.3)	0.006*
	Hospitalization+Home isolation	77(88.5)	10(11.5)	
	Hospitalization	74(74.0)	26(26.0)	
Received Intensive care	No	680(74.2)	236(25.8)	1.000^
	Less than 7 days	5(83.3)	1(16.7)	
	More than 7 days	1(100)	0(0)	
Fever	Mean ± Std Deviation	2.40±1.04	1.89±1.61	<0.001*
Cough	Mean ± Std Deviation	3.56±2.16	2.85±3.19	<0.001*
Shortness of Breath	Mean ± Std Deviation	1.52±1.10	1.01±1.09	<0.001*
Arthritis and Fatigue	Mean ± Std Deviation	2.20±1.46	1.47±1.26	<0.001*
Headache	Mean ± Std Deviation	2.15±1.26	1.73±1.84	<0.001*
Anosmia/Loss of Smell	Mean ± Std Deviation	2.24±1.65	2.05±2.49	<0.001*
Ageusia/Loss of Taste	Mean ± Std Deviation	1.87±1.41	1.63±2.18	<0.001*
Diarrhea	Mean ± Std Deviation	0.54±0.71	0.49±0.69	0.113
Runny Nose	Mean ± Std Deviation	1.08±1.07	1.00±1.44	<0.001*
Insomnia	Mean ± Std Deviation	2.49±1.69	1.54±1.70	<0.001*
Days of treatment	Mean ± Std Deviation	15.43±2.66	13.67±3.92	<0.001*

^Fischer Exact. Other categorical variables tested with Chi-Square

All continuous data were tested with Mann Whitney

*Significant at *P-value* < 0.05

### Collinearity of shortness of breath duration

**Fig 2:**
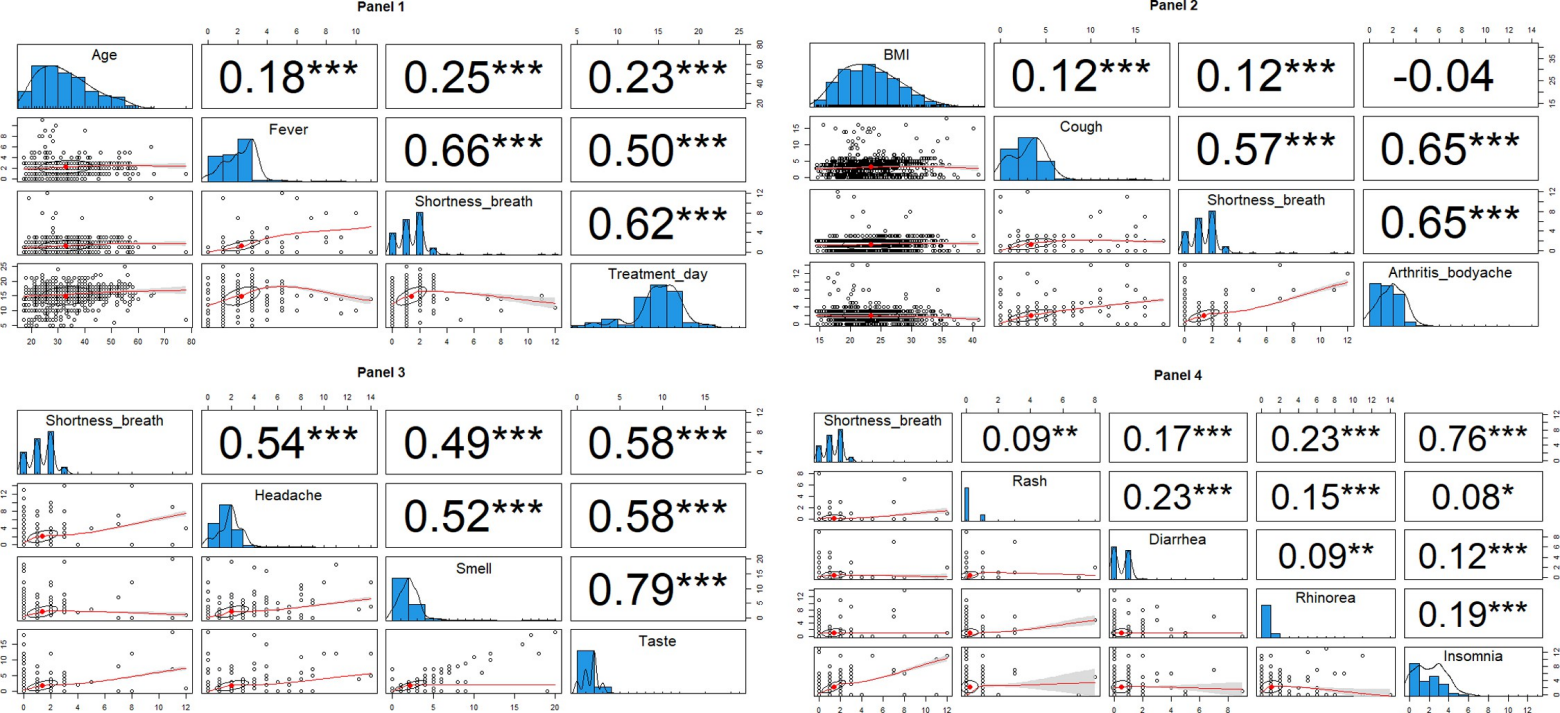
Correlation matrix for multicollinearity analysis.

Tables 1 and 2 show that several significant variables are significantly associated with the overall outcomes. Therefore, addressing one variable representing other factors in reducing the multicollinearity problem in the final statistical analysis is essential. The researchers assume one symptom is associated with other symptoms and the treatment. As COVID-19 affects the respiratory tract, shortness of breath could represent the severity of the disease and is probably linked to other symptoms. The correlation matrix shows depicted in four panels **(Fig 2)** that the total duration of shortness of breath was correlated with other symptoms, age, and body mass index (p<0.001 except with total duration of rash p = 0.005).

Seven demographic factors (p<0.001) except sex (p = 0.600) were associated with shortness of breath duration (Supplement Table 1 in **S1 Table**). In addition, some treatments were associated with shortness of breath (p<0.05) except the prescription of Zinc (p = 0.608), Vitamin C (p = 0.836), Vitamin D (p = 0.242), Favipiravir (p = 0.445) and anticoagulant (p = 0.195) (Supplement Table 2 in **S1 Table**). Hence, the researchers selected the shortness of breath duration as an adjustment factor representing other factors in GEE models.

### All intervention versus post-COVID symptoms in 90 days

In the first model, the researchers evaluated the effect of each exposure (complete vaccination, vitamin C, vitamin D, Zinc, and Favipiravir) in preventing any post-COVID symptoms within 90 days (overall event). An adjustment was made by putting shortness of breath duration as an adjustment factor in binary logistic regression (Supplement Table 3 in **S1 Table**). Administration of anticoagulants was not linked to the shortness of breath duration but was significantly associated with the overall event (p = 0.032 in Table 1). Therefore, the hierarchical analysis also involved anticoagulants as adjustment variables.

Contrary to the bivariate analysis result in Table 1, There was no difference in the occurrence of any post-COVID symptoms in 90 days between fully-vaccinated individuals and incomplete or unvaccinated individuals adjusted by shortness of breath duration (aOR 0.74, 95% CI 0.51–1.08 p = 0.118) (Supplement Table 3A in **S1 Table**). A similar result was found after adding anticoagulants (aOR 0.73, 95% CI 0.50–1.07 p = 0.111) (Supplement Table 3B in **S1 Table**).

Administration of Favipiravir (Supplement Table 3C and 3D in **S1 Table**), Zinc (Supplement Table 3E and 3F in **S1 Table**), Vitamin D (Supplement Table 3G and 3H in **S1 Table**), and Vitamin C (Supplement Table 3I and 3J in **S1 Table**) did not affect post-COVID symptoms in 90 days (p>0.05). However, prolonged shortness of breath duration was associated with post-COVID symptoms in 90 days (average of aOR 1.7 p<0.001).

The combined exposures model shows that people who received vitamin C later than 72 hours after onset/diagnosis showed a protective effect against the overall event (aOR 0.63, 95% CI 0.39–0.99, p = 0.048) compared to participants who received vitamin C less than 24 hours (Table 3), but not other exposures.

**Table 3:** Results of binary logistic regression on post-COVID symptoms in 90 days.

Variable	Subset	B	S.E.	Sig.	aOR	95% CI for aOR	
						Lower	Upper
Shortness of breath duration		.535	.095	.000*	1.707	1.418	2.056
Fully-vaccinated		-.331	.197	.093	.718	.488	1.057
Vitamin_C	<24 hours after onset / diagnosis			.100			
	24–72 hours after onset / diagnosis	.169	.212	.424	1.185	.782	1.794
	>72 hours after onset/diagnosis	-.469	.237	.048*	.626	.393	.996
	Not receiving any treatment	.067	.241	.782	1.069	.666	1.714
Vitamin_D	<24 hours after onset / diagnosis			.819			
	24–72 hours after onset / diagnosis	.161	.223	.469	1.175	.759	1.818
	>72 hours after onset / diagnosis	.233	.300	.437	1.263	.701	2.273
	Not receiving any treatment	.048	.196	.807	1.049	.715	1.540
Zinc	<24 hours after onset / diagnosis			.072			
	24–72 hours after onset/diagnosis	-.154	.229	.501	.857	.547	1.343
	>72 hours after onset / diagnosis	.559	.342	.102	1.749	.895	3.416
	Not receiving any treatment	-.273	.189	.150	.761	.525	1.104
Favipiravir	<24 hours after Diagnosis			.715			
	24–72 hours after Diagnosis	-.083	.304	.784	.920	.507	1.669
	>72 hours after diagnosis	.088	.377	.815	1.092	.522	2.288
	Not receiving any treatment	-.194	.230	.399	.824	.525	1.292
Constant		.635	.274	.020*	1.887		

*Significant at *P-value* < 0.05; aOR = adjusted odds ratio

### Effect of exposures and longitudinal outcomes

The GEE model combining four therapies, and vaccination status, shows that complete vaccination has a protective effect against chronic cough (aOR 0.527, 95% CI 0.286–0.971, p = 0.040 on Supplement Table 4 in **S1 Table**), chronic headache (aOR 0.317,95% CI 0.163–0.616, p = 0.001 on Supplement Table 5 in **S1 Table**), and chronic arthritis (aOR 0.285, 95% CI 0.116–0.703, p = 0.006 on Supplement Table 6 in **S1 Table**). The combination of micronutrient supplementations and Favipiravir gave no significant effect in seven outcomes (Supplement Tables 4–10 in **S1 Table**), adjusted by shortness of breath duration. However, those who did not take Favipiravir showed a protective effect against arthritis (aOR 0.503, 95% CI 0.287–0.882, p = 0.017 on Supplement Table 6 in **S1 Table**). When treating the time of measurement as an interval-censored response, there were no significant changes in the intervention effect on the symptoms. (Supplement Tables 4B-10B in **S1 Table**).

**Fig 3:**
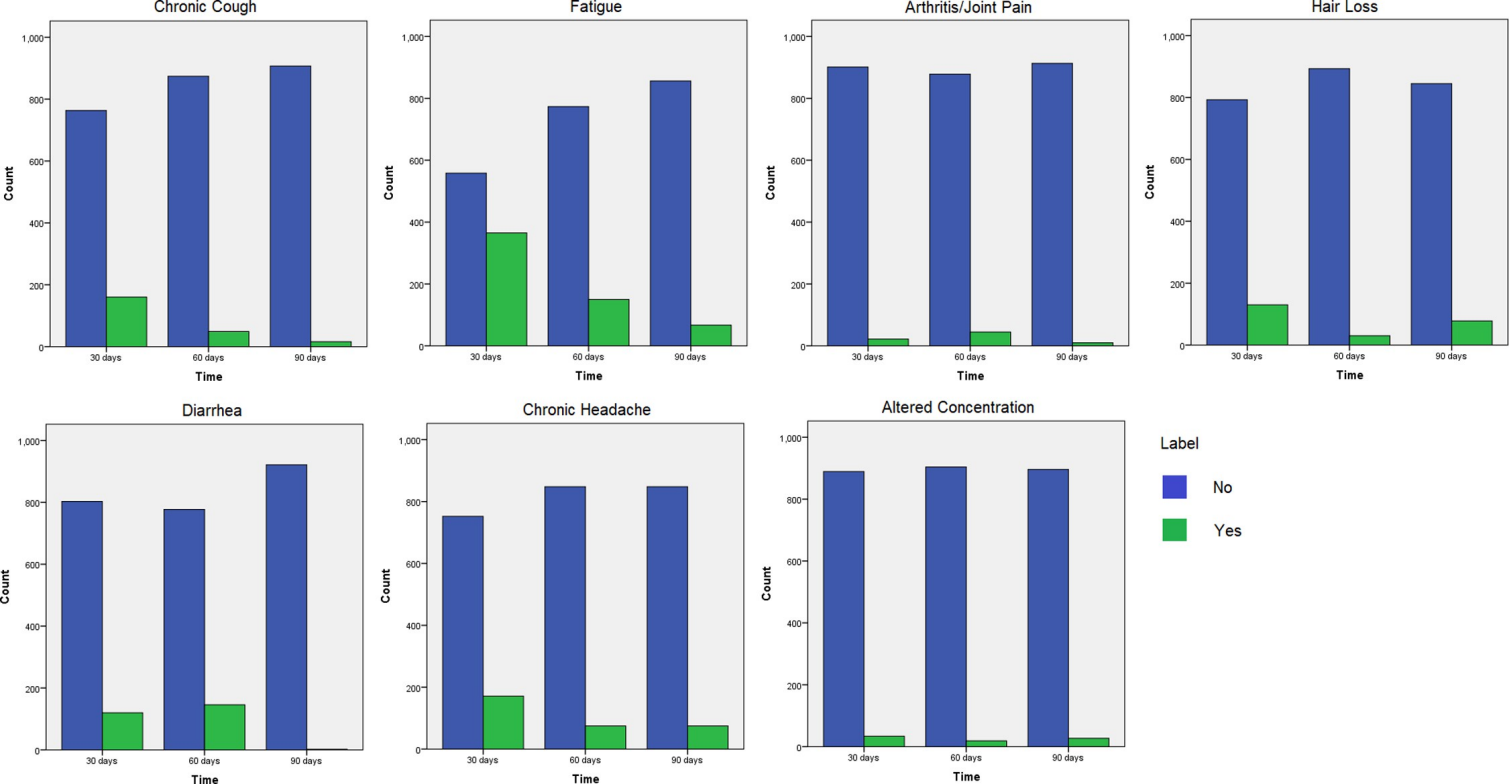
Histogram of post covid symptoms vs. time (30 days, 60 days, and 90 days).

In addition, there was a trend that the symptoms diminished over time (p<0.05 on Supplement Tables 4–10 in **S1 Table**), such as chronic cough, chronic headache, fatigue, and hair loss. However, there was an increased risk on day 60 regarding arthritis (p<0.05) and diarrhea, although it was not significant (p>0.05) compared to day 30. There was no statistically significant difference across time regarding the altered concentration (p>0.05). Fig 3 depicts the histograms of symptoms according to time.

## Discussion

### Self-reported post-COVID symptoms

The terms post-COVID symptoms recently have not been clearly defined. Numerous studies have used various starting points for post-COVID symptoms, including the day of onset, diagnosis, and the day following recovery [5]. This study defined the post-COVID symptoms as any symptoms that occurred following recovery and were counted from the day of onset/diagnosis. As a result, some cases requiring lengthy treatment days were excluded to accommodate earlier post-COVID assessments. Additionally, post-COVID symptoms can occur without any other underlying disorder, and it is critical to rule out this possibility when defining the symptoms as COVID sequelae. The complexity of post-COVID symptoms is also aggravated by unclear screening and diagnosis methods of post-COVID symptoms. Consequently, the study of post-COVID symptoms heavily relies on self-reported symptoms.

This study identified a large percentage of COVID survivors (74.3%) who developed post-any COVID symptoms within 90 days, and these results resemble the finding of the global study [6]. Besides, several symptoms were addressed post-COVID. However, only seven symptoms are discussed in this study as these symptoms are associated with the systemic effect of COVID-19.

As mentioned in the introduction, post-COVID symptoms are associated with CNS involvement and systemic inflammation. Some studies postulate the invasion of the CNS by SARS-COV-2 through hematogenous or neuronal dissemination [7], which irritates the CNS structure and manifests as a headache. In addition, hypometabolism of the CNS [24] and disruption of neurotransmitter regulation [25] are responsible for centrally-regulated fatigue symptoms and cognitive impairment [26]. Cough as a residual symptom is likely due to generalized neuronal hypersensitivity, as proven by radiologic-based studies showing abnormal findings in the brain area responsible for nociceptive processing of cough [27]. The pro-inflammatory cytokines, particularly interleukin 6 (IL-6) released by the injured cells, damage the skeletal muscle and manifest as myopathy [28]. The chemokine increases immune cells’ recruitment, eventually increases the destruction of tissues, and contributes to arthritis [29]. In addition, in the respiratory tract (as the main entry point of SARS-COV-2), pro-inflammatory cytokines (IL-1 β, interferon, or Tumor Necrosis Factor) induce cough and other neuroimmune processes, which eventually decrease the respiratory function [27]. Pro-inflammatory cytokines, particularly interferon-gamma, prematurely stimulate the hair follicle from anagen to catagen stage, where the hair follicle shrinks and the hair separate from its bottom(8), explaining why hair loss occurs more often than 45% of COVID-19 patients [30].

The SARS-COV-2 can be found in stool even after the clearance of the virus in the respiratory tract [8], which could trigger the virus-induced cytopathic effect through Angiotensin-Converting Enzyme 2 (ACE-2) receptors in G.I. cells. The SARS-COV-2 interaction will disrupt the cell function, dysregulate intestinal microbiota [31], and induce an inflammatory response that may affect the lung through the gut-lung axis [32], thus, explaining the G.I. disturbance as one of the post-COVID symptoms.

### Protective effect of exposures in post-COVID symptoms

Sensitivity analysis was executed following two assumptions, post-COVID symptoms do not appear continuously (hence, people may experience it on day 30, unnoticed on day 60, and appears on day 90), or post-COVID symptoms appears without ceasing, and there should be a starting day of the symptoms. It was difficult to determine the exact day when the symptoms appeared in this study. However, when the interval of the event occurrence is known, the interval-censored approach is appropriate. This study applied the logit link function to accommodate the first assumption and complementary log-log for the second assumption. There was no significant interaction between time and intervention. This method strengthened the results of the study.

A previous study showed that fully-vaccinated individuals have protection against COVID-19 of more than 80% [9]. Not only induce immunogenicity, but a study also reported that the mRNA vaccine enhances memory B cells function for six months [33]. Following this assumption, in this study, full vaccination efficacy was 47.3% against chronic cough, 68.3% against chronic headache, and 71.5% against arthritis and remained consistent when treating the outcomes as interval censored-response (45.8%, 66.3%, and 60.9%, respectively). In addition, shortness of breath duration, which indicates the disease severity, was significantly shorter in the fully-vaccinated group (0.85 versus 1.51 average days p<0.001). However, in the GEE model of each symptom, there was no significant association of shortness of breath duration with post-COVID (except in altered concentration), suggesting that vaccination plays a different role in preventing post-COVID. Viral load is not linear with the severity of disease [34], and there is a strong indication that fully-vaccinated individuals have a different response to viral load at an earlier stage than unvaccinated. A prospective study in the United Kingdom shows that fully-vaccinated people have a faster viral load decline than unvaccinated [35]. The faster viral load clearance reduces the further dissemination of the virus to other system organs such as CNS, thus preventing direct injury of tissues by SARS-COV-2 and the residual symptoms.

This study focused on Favipiravir as the primary antivirus. Favipiravir dosage in COVID-19 is based on the dose of another influenza where the initiation dose ranges from 1600–1800 mg per 12 hours for the first day, followed by 600–800 mg twice daily. This variation exists in some national guidelines [36], and Indonesia follows the lower initiation dose. Favipiravir outperforms other antiviruses, including Oseltamivir (20), despite the various initiation and maintenance doses, particularly in mild-moderate cases. Molnupiravir has been on the list of preferred treatments for COVID since January 2022, specifically for the non-severe case with a higher risk of hospitalization [37], which is why this study was unable to assess this medication.

Vitamins and minerals have significant roles in immunity. Vitamin C exhibits an immunomodulatory effect by enhancing the barrier activity of the epithelial cell, phagocytosis activity, microbial killing, differentiation, and proliferation of lymphocytes, and modulating cytokine production [38]. Vitamin D is also involved in innate and adaptive immunity through its receptor on the cells [39]. A higher level of vitamin D is linked to a higher level of cathelicidin, an antimicrobial peptide associated with a lower level of lung injury [40]. Inhibition of ACE2 expression is also promoted by zinc and vitamin C, thus reducing the cell’s susceptibility to SARS-COV-2 [41]. Zinc demonstrates inhibition to RdRp activity, similar to the antiviral drug mechanism. Moreover, Zinc inhibits nuclear factor‐kB (NF‐kB), essential for expressing inflammatory cytokines. Zinc is essential in maintaining airway epithelial and thus preventing lung injury [42].

Despite the plausible mechanism to interfere with post-COVID symptoms, Favipiravir and other medications showed an inconsistent effect on post-COVID symptoms. However, not taking Favipiravir was associated with a lower probability of arthritis (Supplement Table 5 in **S1 Table**) than those who took Favipiravir < 24 hours after diagnosis. Therefore, there is an assumption that Favipiravir could cause arthritis. In addition, a study reveals that Favipiravir induced hyperuricemia [43] and gouty arthritis in COVID-19 patients [44]. However, this study could not confirm this assumption as no examination of uric acid was conducted. Also, the authors cannot rule out the possible joint inflammation contributing to chronic arthritis.

Delaying vitamin D administration was also connected to arthritis in this study. Participants who took vitamin D more than 72 hours after diagnosis/onset had a higher risk of developing arthritis than those who initiated the treatment within 24 hours, although there was no risk difference between those who did not take it and early initiation (Supplement Table 5 in **S1 Table**). Aside from having anti-inflammatory properties and stimulating the immune system, vitamin D also induces chondrocyte hypertrophy [45] which counters cartilage destruction, mainly due to extracellular inflammation.

Significant changes over time (p<0.001) were observed in fatigue, arthritis, chronic cough, and chronic headache, where the tendency to develop the symptoms was lower (except for hair loss). In addition, there was a significant increase in diarrhea in 60 days. It is unclear whether the inflammation targets the G.I. tract or becomes an infection source. However, the higher affinity of SARS-COV-2 to the ACE2 receptor in G.I. cells is the underlying reason for the altered physiological function of the G.I. tract, including Dysbiosis that leads to diarrhea [46]. The SARS-COV-2 could be found in the G.I. tract and last for several weeks(47), and the prolonged Dysbiosis may affect the G.I. tract and other systems related to the gut lung axis [47]. This theory could be the plausible reason why "late" post-COVID symptoms occur.

When medications showed insignificant effects, confounders might play roles in the post-COVID symptoms. Therefore, this study retrieved detailed information on demography and comorbidities as these factors may act as mediators or moderator variables. Most demographic factors were not associated with symptoms within 90 days except for those living alone (Table 1). Additionally, these factors were connected to the shortness of breath duration, which denotes the severity of the disease. Hence, the severity of the disease would act as a moderator variable between demographic factors and post-COVID symptoms.

Nevertheless, demographic factors could be a mediator variable as these factors are associated with physiological aspects. Stress, anxiety, and depression, either due to external stressors, including strict isolation, loss of productivity, and inability to perform the exercise, could contribute to chronic fatigue [48] and other psychologically-related post-COVID outcomes, thus, explaining why the effect of medication was insignificant. Therefore, it is essential to address the psychological situation of people who underwent home isolation alone and support this group emotionally.

### Concern in methodology

This longitudinal study covered the period when delta variants were dominant in Indonesia. However, other variants such as Alpha and Beta were found in the western part of Indonesia during that period. Furthermore, this study could not capture the surge of omicron cases as it dominated Indonesia at the beginning of 2022, whereas all participants were contracted before that.

The questionnaire demanded the participants to confirm that their symptoms were not due to other underlying causes, such as performing a radiology assessment or spirometry for chronic cough or attention test to confirm deterioration in concentration. However, the authors acknowledge that the clinical confirmation of the self-reported outcomes might not be feasible. Furthermore, standard clinical confirmation might not capture the clinical changes reported by participants, and complex modalities may capture the lung abnormalities in Long COVID patients, such as Xenon Magnetic Resonance Imaging [49] which is not a routine procedure in clinical practice. In addition, clinical questionnaires might introduce survey fatigue, as mentioned in the methodology, and a lack of supporting studies assessing the clinical questionnaire’s reliability in COVID-19.

Vaccination status does not provide similar protection. It is essential to measure the antibody level as some evidence regarding the waning efficacy of vaccines has emerged [50]. In this study, between July 2021-early November 2021, when most participants entered the cohort, the percentage of fully vaccinated people in Indonesia ranged from 5%-26.8%, and this cohort recorded an average of 18.2%. Hence, it was not feasible to evaluate the effect of the booster vaccine, heterologous regimen, or even mRNA vaccine in this study as during that time; these policies were not yet implemented.

This study also identified possible heterogeneity in micronutrient supplementation. Although the guideline states a specific dose and frequency, some participants preferred combined micronutrient tablets where the doses might differ from the recommendation. The zinc level in these combined tablets is lower than the recommended dose (around 10 mg). Participants might find difficulties distinguishing the vitamin D3 (Cholecalciferol) and Vitamin D2 (Ergocalciferol), and there are no clear sentences in the guideline for which vitamin D is recommended. When the participants took lower than the recommended dose, they tended to answer "not taking any medication," thus would underestimate the effect of taking a lower dose of micronutrient supplementation. The researcher also acknowledged that medication adherence could be an unobserved latent variable.

## Conclusion and recommendation

This study addresses the importance of full vaccination to reduce post-COVID symptoms (particularly headache, chronic cough, and arthritis) by enhancing viral clearance and preventing virus dissemination to other system organs. It is recommended to reconsider micronutrient supplementation as part of therapy and simplify the COVID-19 treatment guideline. Furthermore, Favipiravir has conflicting effects on post-COVID symptoms. An indication that a lower dose of Favipiravir in Indonesia’s guideline is related to this finding, although further study is needed. Furthermore, Favipiravir should be carefully given to patients with hyperuricemia. Finally, the authors recommend mental support to those who live alone during home isolation as some post-COVID symptoms are linked to the psychological condition.

## Supporting information

S1 Table Supplementary tables. (XLSX)
